# dGTP Starvation in *Escherichia coli* Provides New Insights into the Thymineless-Death Phenomenon

**DOI:** 10.1371/journal.pgen.1004310

**Published:** 2014-05-08

**Authors:** Mark Itsko, Roel M. Schaaper

**Affiliations:** Laboratory of Molecular Genetics, National Institute of Environmental Health Sciences, Research Triangle Park, North Carolina, United States of America; University of Illinois at Urbana-Champaign, United States of America

## Abstract

Starvation of cells for the DNA building block dTTP is strikingly lethal (thymineless death, TLD), and this effect is observed in all organisms. The phenomenon, discovered some 60 years ago, is widely used to kill cells in anticancer therapies, but many questions regarding the precise underlying mechanisms have remained. Here, we show for the first time that starvation for the DNA precursor dGTP can kill *E. coli* cells in a manner sharing many features with TLD. dGTP starvation is accomplished by combining up-regulation of a cellular dGTPase with a deficiency of the guanine salvage enzyme guanine-(hypoxanthine)-phosphoribosyltransferase. These cells, when grown in medium without an exogenous purine source like hypoxanthine or adenine, display a specific collapse of the dGTP pool, slow-down of chromosomal replication, the generation of multi-branched nucleoids, induction of the SOS system, and cell death. We conclude that starvation for a single DNA building block is sufficient to bring about cell death.

## Author Summary

Starvation of cells for DNA precursor dTTP is strikingly lethal in many organisms, like bacteria, yeast, and human cells. This type of death is unusual in that starvation for other nutritional requirements generally results in growth arrest, but not in death. The phenomenon is called thymineless death (TLD), because it was first observed some 60 years ago when a thymine-requiring (*thyA*) *E. coli* strain was exposed to growth medium lacking thymine. The TLD phenomenon is of significant interest as it is the basis for several chemotherapeutic (anticancer) treatments in which rapidly growing cells are selectively killed by depletion of the cellular dTTP pool. The precise mechanisms by which cells succumb to dTTP depletion are of significant interest, but have remained elusive for a long time. In the present work, we demonstrate for the first time that the effect is not specific for dTTP starvation. We show that an *E. coli* strain starved for the DNA precursor dGTP dies in a manner similar to dTTP-starved cells. The effect, which we have termed dGTP starvation, might be exploited - like TLD - therapeutically.

## Introduction

Starvation of cells for the DNA precursor dTTP can cause rapid cell death in all domains of life [1]. This phenomenon, called thymineless death (TLD), was first discovered in 1954 in *E. coli* upon exposing thymine-requiring (*thyA*) strains to medium lacking thymine [2]. As TLD can be promoted in cells from bacteria to man, it has been widely employed for therapeutic purposes. Methotrexate and trimethoprim, both antifolates, and 5-fluorouracil are antitumor and antibacterial agents based on their ability to block thymidylate (dTMP) synthesis [3], [4] leading to low dTTP levels that kill or prevent proliferation of actively-dividing cells. However, despite decades of interest, our understanding of the TLD phenomenon is still incomplete, particularly with regard to the primary initiating events that cause cell death. Recent progress has revealed a complexity of participating and contributing events, and has led to models centered on the impairment of DNA replication and resulting stalling of replication forks [5]–[9]. Such stalled forks give rise to DNA breakage if not repaired by homologous recombination. Importantly, despite the stalling of existing replication forks, initiation of new replication forks at the oriC chromosomal origin can continue [9], [10], causing increased complexity of the chromosome, which becomes a major determinant of cell death [7], [9]. Recombinational processes play an important role throughout TLD in at least a dual fashion: they can rescue starving cells from early stages of TLD, but ultimately contribute actively to death at later stages by creating unresolvable or unrepairable intermediates and DNA breaks [5], [8]. Notably, significant breakage and disappearance of origin-containing DNA is observed, consistent with the importance of ongoing DNA initiation in TLD [6], [9]. TLD is also accompanied by persistent SOS induction, which contributes to cell death by initiating lethal filamentation [5].

However, a critical unanswered question is whether the phenomenon is truly thymine-specific or can be, likewise, imposed by starvation for other DNA precursors. Obviously, models based on stalled DNA replication would apply equally if stalling were mediated by starvation for any other dNTP. However, selective manipulation of the concentration of individual dNTPs is experimentally difficult, because their joint *de novo* synthesis is regulated by feedback on the enzyme ribonucleotide reductase [11]. Until now, dTTP was the only nucleotide for which the phenomenon could be demonstrated, as its pool can be manipulated separately through the thymine salvage pathway [12].

Nevertheless, long-sought conditions by which cells can be starved specifically for a dNTP other than dTTP were found, serendipitously, in our studies of the *optA1* allele of the *E. coli dgt* gene. The *dgt* gene encodes a dGTPase with an unusual activity, hydrolyzing dGTP into deoxyguanosine and triphosphate (PPP_i_) [13], [14]. Its deletion was found to result in a spontaneous mutator effect [15], which was attributed to possible changes in the cellular dNTP levels, particularly dGTP. Indeed, an approximately 2-fold increase in the dGTP level of a *dgt* mutant had been described [14]. The *optA1* allele of *dgt* is a promoter-up mutation, which increases gene expression by as much as 50-fold [16]. Consistent with this up-regulation a decrease in dGTP level was reported [17], although modest in view of the 50-fold gene overexpression. This presumably reflects the ability of cells to adjust their dNTP levels through feedback regulation on the ribonucleotide reductase. In the present study we report that a more dramatic and specific dGTP decrease can be achieved by combining the *optA1* allele with a defect in the *gpt* gene. The *gpt* gene functions in purine salvage by converting guanine into guanosine monophosphate (GMP). In the present study, we show that an *optA1 gpt* strain grown in minimal medium with casamino acids (CAA) in the absence of an external purine source, like hypoxanthine (Hx) or adenine, dies in a manner sharing many of the features associated with TLD. Certain differences with TLD are also noted, which we argue reflect different kinetic manifestations of the same intrinsic mechanism. We propose to term this phenomenon dGTP starvation.

## Results

### Impaired growth of an *E. coli optA1 gpt* strain

**Figure 1:**
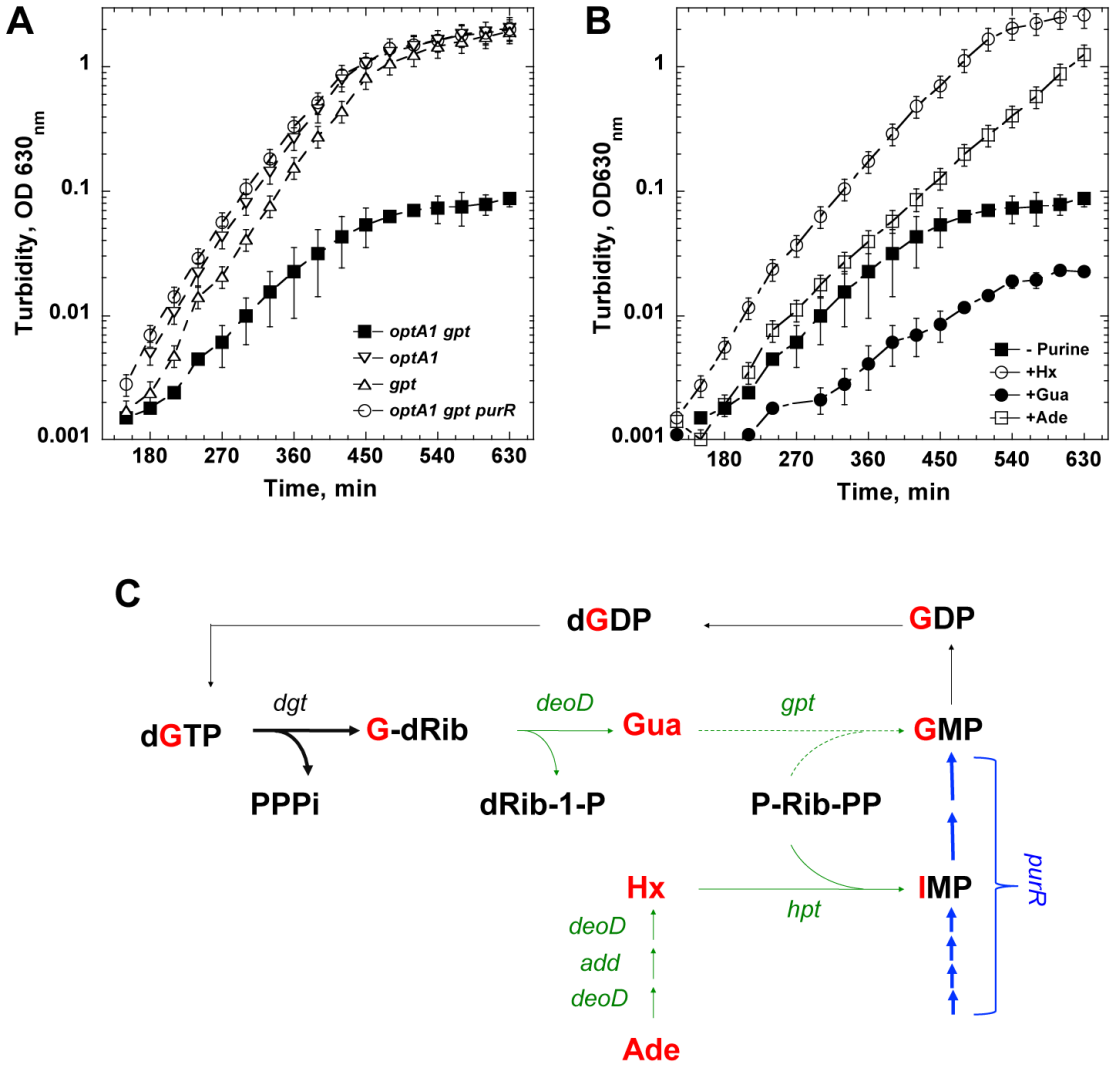
Defective growth upon purine starvation. (**A**) Growth defect of an *optA1 gpt* strain upon culturing in minimal glucose medium enriched with casamino acids without added purine source. Several controls are also shown (see text for details). Error bars are from three different measurements. (**B**) Complementation of the growth defect of an *optA1 gpt* strain by hypoxanthine (Hx), adenine (Ade), but not guanine (Gua), added at 50 µg/ml each. The cultures were started from a 5,000-fold dilution of an overnight stationary culture grown in the presence of hypoxanthine. (**C**) Relevant metabolic pathways for *de novo* synthesis and salvage of guanine and guanine nucleotides, illustrating how the *optA1 gpt* combination may become starved for dGTP (see text for details). One alternative pathway for synthesis of GMP from Gua that is not indicated is the conversion of Gua to guanosine by the DeoD purine nucleoside phosphorylase by condensation with Rib-1-P followed by conversion of guanosine to GMP by guanosine kinase (*gsk* gene product). However, this pathway for GMP synthesis is not very efficient [52]. The gene symbols are: *dgt* - dGTP triphosphohydrolase; *deoD* - purine nucleoside phosphorylase; *gpt* - guanine phosphoribosyltransferase; *hpt* - hypoxanthine phosphoribosyltransferase; *add* – adenosine deaminase; *purR* – purine repressor (transcription factor controlling *de novo* synthesis of purine nucleotides) [21]. Green and blue – salvage and *de novo* synthesis pathways, respectively. Arrows are as follows: thin – wild-type enzyme levels; thick – elevated levels of dGTP triphosphohydrolase (*optA1* - *dgt* up-promoter) or of enzymes of the PurR regulon (*purR* deletion strain); dashed - lack of activities in a *gpt* deletion strain. Gua – guanine; Hx – hypoxanthine; Ade – adenine; dG-Rib – deoxyguanosine; IMP – inosine monophosphate; PPP_i_ – tripolyphosphate; dRib-1P – deoxyribose-1-phosphate; P-Rib-PP – 5′-phosphoribosyl-1-pyrophosphate (PRPP).

The initial observation that triggered our interest was that the *optA1* allele of *dgt* caused impaired growth when the strain also contained the large (120-kb) Δ(*pro-lac*)_X111_ chromosomal deletion [18]. When stationary cultures of such a strain were diluted by at least 5,000-fold in minimal glucose medium enriched with CAA (1%), the cultures failed to grow beyond OD_630 nm_ = 0.1. Complementation of the deletion by an F'*prolac* covering the deleted region reverted the cells to normal growth. Upon reconstruction of this defect in the widely-used MG1655 strain background, we found that the growth impairment was attributable to the combination of *optA1* with the lack of *gpt*, a gene located inside the boundaries of the Δ(*pro-lac*)_X111_ deletion. The *gpt* gene encodes Guanine Phosphoribosyltransferase, a purine salvage enzyme responsible for salvaging guanine, hypoxanthine, and xanthine via their conversion to the corresponding NMP, with guanine being the preferred substrate due to the lowest *K_m_* [19]. The growth curves displayed in Fig. 1A show the growth defect of the *optA1 gpt* strain. While the single *optA1* or *gpt* strains show normal growth in the minimal medium with casamino acids, the *optA1 gpt* double mutant fails to reach beyond OD_630 nm_ = 0.1 for at least 10 hrs. In contrast, the double mutant strain grows normally in the presence of the added purine sources hypoxanthine (Hx) or adenine (Ade), as shown in Fig. 1B. Addition of guanine (Gua) as purine source has no such effect; in fact, it exacerbates the growth defect.

The deleteriousness of the *optA1 gpt* combination may be understood based on the activities of the corresponding Dgt and Gpt enzymes within the salvage and *de novo* purine biosynthesis pathways. Diagram 1C shows how enhanced dGTPase activity resulting from *optA1* leads to increased breakdown of dGTP, yielding deoxyguanosine (G-dRib) and deoxyribose-1-phosphate (dRib-1-P) and, subsequently, guanine (Gua) upon further metabolism by the DeoD purine nucleoside phosphorylase. In the *gpt* background, this guanine cannot be readily returned back, via GMP, to the guanine nucleotide pool; therefore, the expected result is a limitation for purine nucleotides, presumably most acutely for dGTP. Consistent with this model are the observed alleviation of the growth inhibition by addition to the growth medium of exogenous purines like hypoxantine (Hx) or adenine (Ade), which do not require *gpt* action (Fig. 1C), but not guanine (Gua), which is, in fact, inhibitory (see Fig. 1B). Any accumulated guanine is expected to contribute further to the starvation, as it is a corepressor for the PurR repressor [20], [21] controlling *de novo* purine biosynthesis (Fig. 1C). Indeed, we found that one alternative way of circumventing the *gpt* block (see Fig. 1C) is the stimulation of *de novo* IMP production by inactivation of the *purR* repressor [21], as shown in Fig. 1A.

**Figure 2:**
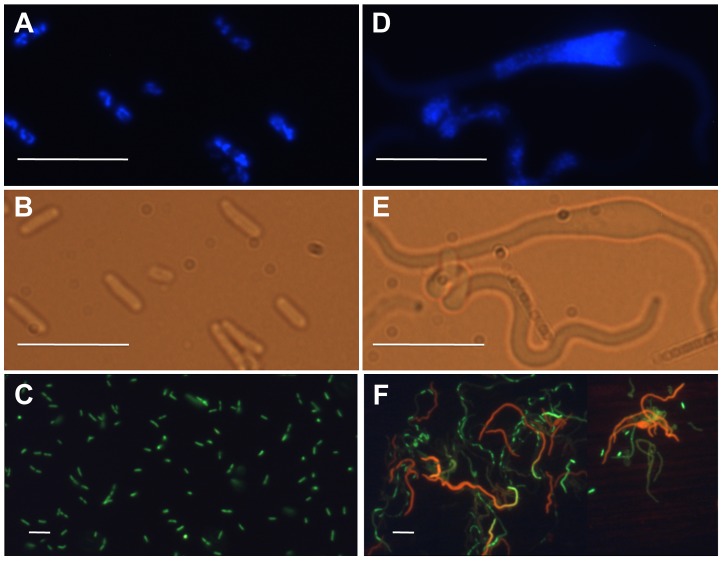
Fluorescence and phase-contrast micrographs of an *optA1 gpt* strain grown in the presence (A, B, C) or 7 hours in the absence (D, E, F) of hypoxanthine. (A, D) Nucleoid morphology (DAPI stain), (B, E) cell morphology, and (C, F) Life (green)/Dead (red) staining. Bar – 10 µm.

Cell and nucleoid morphology of *optA1 gpt* strains were followed by microscopy, as shown in Fig. 2. The starved cells developed progressively extensive filamentation with swollen regions (bulges) (Fig. 2D and E) in the middle of the cells. At the 7 hr time point, DAPI staining revealed disturbed nucleoid shapes within the filaments, which became fused and compacted (Fig. 2D). The enlarged nucleoids coincided with the filament bulges, thus accounting for the distortion of the cell envelopes. Use of Live/Dead staining indicated extensive death of the filaments (Fig. 2F).

**Figure 3:**
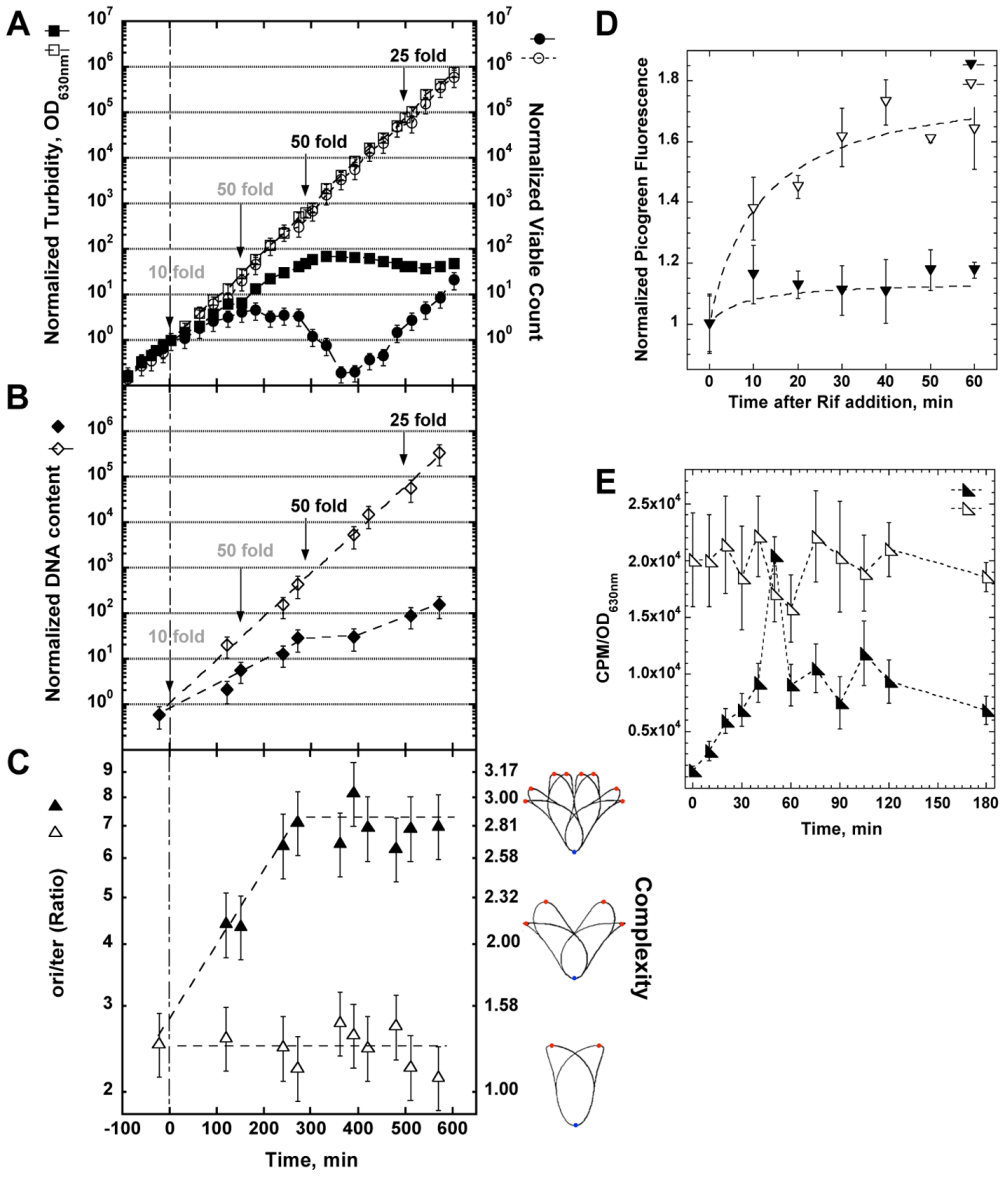
Physiological changes in *optA1 gpt* strain during growth in medium without hypoxanthine (Hx). (**A**) Cultures of the *optA1 gpt* strain were grown exponentially in the presence of hypoxanthine (Hx) (50 µg/ml). At time 0 (OD_630 nm_ = 0.1), two aliquots were filtered and diluted 10-fold into identical fresh, prewarmed medium with or without Hx. During subsequent growth, cells were kept in active growth stage by dilutions, when necessary, to keep the OD_630 nm_ below 0.2 at all times. Such dilutions are indicated by arrows. Dilutions applied to both cultures are in gray; those applied to only the +Hx culture are in black. All OD_630 nm_ and viable count values displayed on the y-axes are normalized to the initial value at t_0_ and adjusted for the applied dilutions. The values for OD_630 nm_ (turbidity) and viable count after the initial 10-fold dilution (at time zero) were chosen as reference point for all comparisons (10^0^ = 1). The actual values at this time point were 0.01 for the OD_630 nm_ and 2.5×10^6^ cells per ml for the viable count. Turbidity (OD_630 nm_) is represented by squares, viable count by circles. Open symbols represent growth with Hx; closed symbols, growth without Hx. (**B**) DNA content of *optA1 gpt* cells growing with and without Hx. Details as in (A) above. The DNA content value for the zero time point is 60 ng/ml. Open symbols, growth with Hx; closed symbols, growth without hypoxanthine. (**C**) Determination of ori/ter ratio of *optA1 gpt* strain (left scale) or translated into complexity 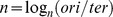 (right scale). Open triangles, growth with Hx; closed triangles, growth without Hx. The illustrations to the right of the complexity scale are adapted from the Cell Cycle Simulation Program (http://simon.bio.uva.nl/cellcycle/); the illustrations demonstrate different levels of nucleoid structure (i.e., chromosomes with 1, 2 or 3 fork positions), which are modeled to progressively appear during purineless growth. The red dots represent the chromosomal replication origin (oriC). (**D**) Measurement of run-out DNA synthesis during rifampicin treatment of cultures containing Hx (open triangles) or during dGTP starvation (closed triangles). Hx was removed at time zero. (**E**) Measurement of DNA synthesis rate. *optA1 gpt* cultures either containing hypoxanthine (open triangles) or starved for hypoxanthine (closed triangles) for different times were subjected to a 3-min pulse with [methyl-^3^H]-thymidine (see Materials and Methods). Counts per minute (CPM) were normalized relative to turbidity of the corresponding culture, as this parameter approximately reflects the amount of DNA (compare A and B). For all panels (A through E) standard deviations (error bars) were calculated from three experiments.

Subsequent experiments were designed to measure more carefully the physiology of the starving cells. It soon became clear that the growth defect of the *optA1 gpt* strain was dependent on the cell density: only strongly diluted cultures (for example 1/5,000 from overnight cultures) showed the growth restriction. This suggested to us that maintenance of an active growth phase was required; reduced growth experienced at higher biomass values would allow the cells to escape. On that basis, we developed a standardized protocol in which inoculates from overnight cultures were first grown for a sufficient number of generations in the presence of hypoxanthine to assure exponential growth, and then cells were transferred to medium without hypoxanthine. When necessary, subsequent dilutions were made in fresh medium to keep the OD_630 nm_ at or below 0.2. Performed in this manner, the experiment of Fig. 3A shows that non-starved cells are able to continue in exponential growth indefinitely, as expected. However, cells growing without hypoxanthine display a slowdown in biomass growth (OD) rate compared to the control and an eventual complete growth arrest at 5–6 hrs. More importantly, the viable count of the starved culture increased initially only by about 4-fold overall and then declined by 40- to 50-fold, indicating extensive death of the cells.

Interestingly, after the 6 hr time point the culture appeared to recover (Fig. 3A). As discussed later in more detail, this recovery reflects the accumulation of suppressor mutants that have become resistant to the starvation condition. However, we will first report on the properties of the starving and dying *optA1 gpt* cells prior to the appearance of the suppressors. The Supplementary Fig. S1 shows the importance of the 50-fold dilution at the 150 min time point; lower dilutions are not sufficient.

### DNA synthesis in starving cells

In addition to turbidity and viable cell count, we also monitored the extent of DNA synthesis in the starving cells. The results in Fig. 3B show that the starved cells synthesized DNA at a reduced rate. Much of this reduction may reflect a slow down of ongoing replication forks due to the shortage of dGTP, as further explored in the following three experiments.

In the experiment of Fig. 3C we followed the status of the bacterial chromosomal DNA by determining the ratio of chromosomal origins to termini (ori/ter) by Q-PCR. The ratio was around 2.6 in the non-starved cells, but it increased progressively to a value above seven during hypoxanthine starvation. Such increase is consistent with a slowed-down progression of the replication forks (increase in chromosomal replication time or *C*-time) along with a continued production of new forks at the *oriC* origin. This is also one of the hallmarks of thymine starvation, at least in the early stages of the process [7], [9], [10]. Informatively, the ori/ter ratio can be used to predict the branching (complexity) of the nucleoid [22] under these conditions. As shown in Fig. 3C (right side), the chromosomes of the starved cells are predicted to become increasingly complex.

We also investigated the progress of existing replication forks in the absence of new initiations. For this, we used the antibiotic rifampicin, which permits, in principle, continuation of DNA synthesis from existing forks but prevents new initiations [23]. The results in Fig. 3D show that the non-starved cultures display the saturation kinetics of DNA synthesis typically observed in this kind of experiment (replication fork run-out). In contrast, the hypoxanthine-starved cultures show a dramatic loss of DNA synthesis capacity (to roughly only 15% of the control), consistent with the predicted sudden limitation in dGTP upon hypoxanthine removal. It appears that, in the absence of transcription, even existing forks cannot be completed. The simplest explanation would be a near complete loss of dGTP upon hypoxanthine deprivation under these conditions of the inhibited transcription.

To more directly investigate the rates of DNA synthesis in starved vs. non-starved cells, we conducted pulse-labeling experiments using [methyl-^3^H]-thymidine. The results shown in Fig. 3E indicate that the hypoxanthine-starved culture suffers from an immediate about 20-fold reduction in the DNA synthesis rate. Interestingly, within the next 40 minutes, DNA synthesis capacity recovers slowly to approximately 50% of the control value, presumably due to transcriptional adaptation that may recover dGTP levels, at least in part. The near-complete loss of DNA synthesis capacity at the earliest time point is fully consistent with the inability of the cells to complete their ongoing forks in the presence of rifampicin (see Fig. 3D). These results are also supported by the changes in dGTP level as described in a subsequent section.

The Fig. 3E also shows the appearance of a spike of thymidine incorporation about 50 minutes after the start of starvation. This spike does not represent an occasional fluctuation within the measurements, as it was observed reproducibly in at least four repeated experiments. The simplest explanation for this spike may be an initiation event occurring at this time point. If correct, the interesting question arises as to how the present circumstances can lead to a coordinated culture-wide event. Following the spike, DNA synthesis capacity quickly drops to the pre-spike level. This inability to sustain the new higher rate of [methyl-^3^H]-thymidine incorporation indicates that, regardless of the number of forks, the total amount of DNA synthesis is severely constrained, likely due to the amount of available dGTP (see section below).

### Depletion of the dGTP pool

**Figure 4:**
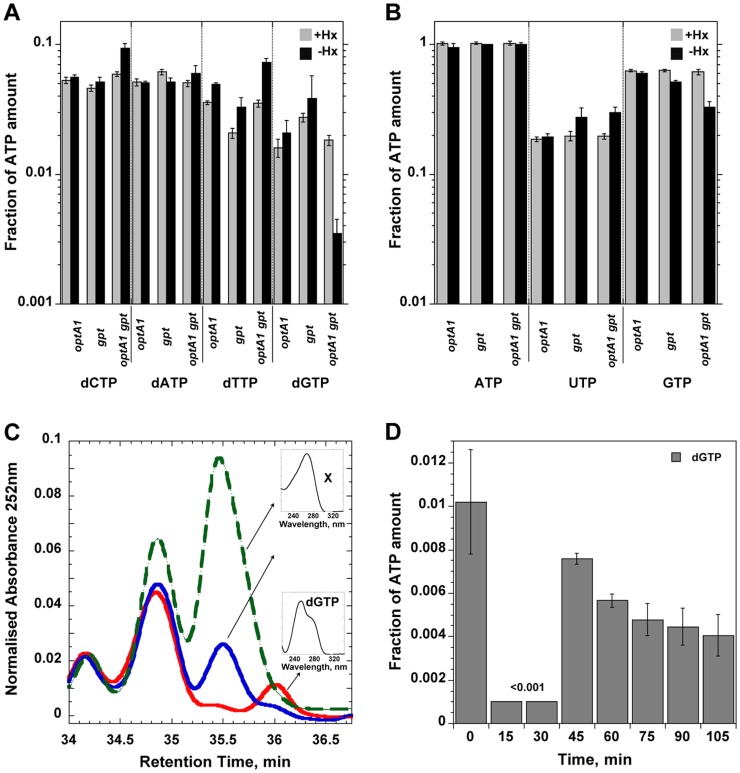
Nucleotide pool effects in Hx-starved strains. Shown are (**A**) dNTP and (**B**) NTP pools in strains grown with (grey) or without (black) hypoxanthine (Hx) at two hours after withdrawal of Hx in the indicated strains. Nucleotide amounts were normalized relative to the ATP peak as in [38]. The amount of ATP calculated as ATP/(OD_630 nm_×sample volume) was largely unchanged among each of the strains. CTP could not be quantitated in these experiments. Standard deviations (error bars) were calculated from three experiments. (**C**) Chromatogram showing disappearance of dGTP during Hx starvation of the *optA1 gpt* strain. The red line represents growth in presence of Hx; the blue line and green lines correspond to 2 and 4 h of Hx starvation, respectively. The small inserts show the absorption spectra of dGTP and the unknown substance (X) that appeared during the starvation and interfered with quantitation of the dGTP at later time points. (**D**) Time course for dGTP pool changes in hypoxanthine-starved *optA1 gpt* strain. Cells were grown in hypoxanthine-containing medium (+Hx). At t = 0, hypoxanthine was withdrawn, and samples were withdrawn at indicated times. The HPLC analysis protocol was modified to provide for improved resolution of the dGTP peak (see Materials and Methods). The dGTP level at 15 and 30 min of starvation was below the detectable limit of the method (0.001).

To investigate the effects of the starvation on the dNTP DNA precursors, we analyzed the intracellular dNTP pools after 2-hr and 4-hr incubations in the purineless medium. A clear ∼6-fold reduction, was seen in the dGTP concentration at 2 hrs (Fig. 4A, Table S1), while the concentration of the other dNTPs was not significantly affected (except for a possible small increase in the dCTP and dTTP pools). At times later than 2 h, dGTP could no longer be detected, although this was due in part to the appearance of another, as yet unidentified peak in the HPLC profile nearby the dGTP peak (See Fig. 4C). No changes in dGTP level were noted in the single *optA1* or *gpt* mutants. In contrast, only a slight reduction in the rGTP pool was noted (2-fold or less, see Fig. 4B, Table S1).

To investigate the time course of dGTP depletion upon Hx removal we withdrew samples at a series of earlier time points and analyzed them by HPLC by a slightly different protocol designed to improve the resolution of the dGTP peak. During the first 30 minutes after hypoxanthine withdrawal, the dGTP pool was dramatically reduced (Fig. 4D); in fact, no dGTP could be detected above the (0.001) detection limit, indicating that the dGTP level was decreased by 10-fold or more. Interestingly, the dGTP level temporarily recovered at the 45-min time point but declined afterwards. We note that the kinetics of dGTP reduction of Fig. 4D are fully consistent with the DNA synthesis rate changes (Fig. 3E). Thus, DNA synthesis in the *optA1 gpt* mutant appears strictly governed by dGTP availability.

### SOS induction in starved *optA1 gpt* strains

**Figure 5:**
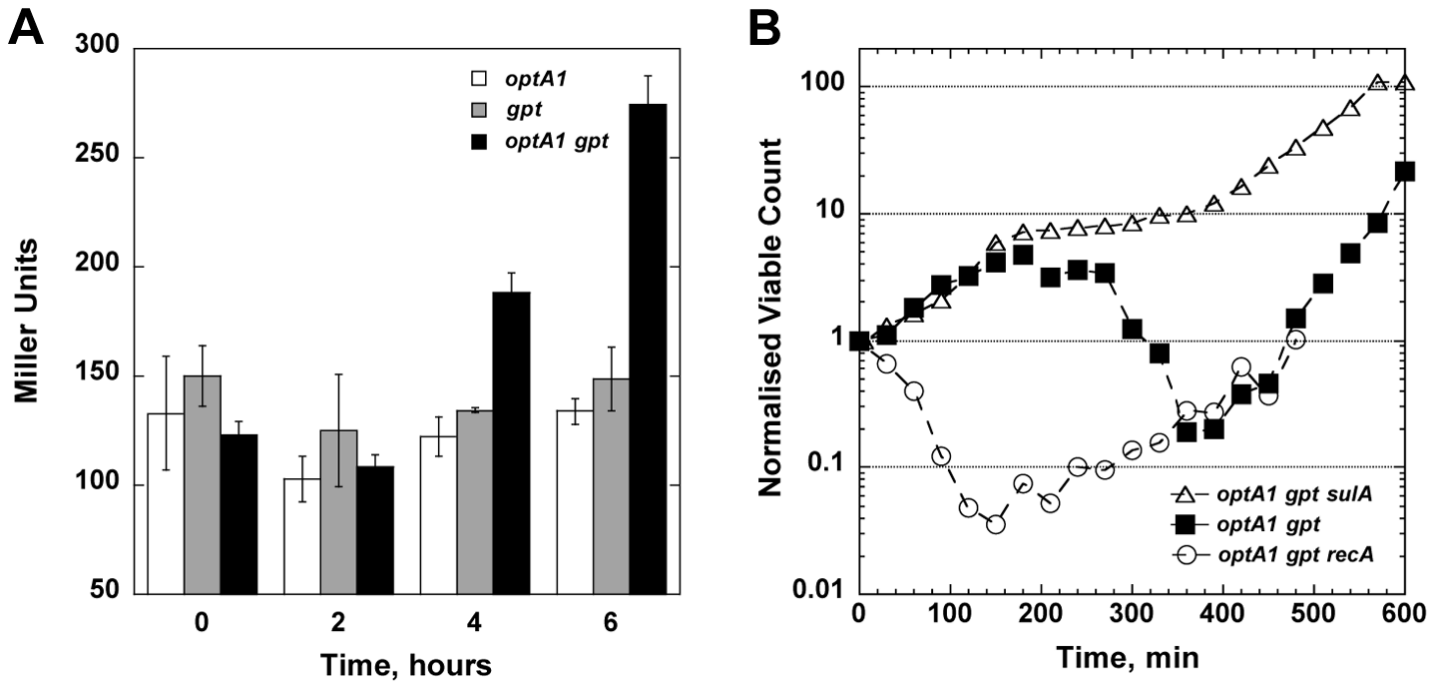
Role of the SOS response in dGTP-starved cells. (**A**) Induction of the SOS system upon hypoxanthine (Hx) starvation. Cultures of *optA1*, *gpt*, and *optA1 gpt* strains containing a plasmid-carried SOS reporter gene (*umuC*::*lacZ*) were grown in minimal medium (with 1% casamino acids) in the absence of Hx for 2, 4, and 6 hours, and samples were processed for liquid β-galactosidase assay. (**B**) Effect of *recA* and *sulA* deletions on cell growth during dGTP starvation. Shown are the normalized viable counts (see Legend to Fig. 3) of the *optA1 gpt* (filled squares), *optA1 gpt recA* (open circles), and *optA1 gpt sulA* strains (open triangles) during Hx starvation initiated at time zero.

One of the hallmarks of TLD is the occurrence of significant DNA damage and induction of the SOS response [5], [8]. To investigate whether cell death during dGTP starvation is also accompanied by SOS induction, we assayed the expression level of an *umuC*::*lacZ* reporter construct, which can be used as a diagnostic for induction of the damage-inducible SOS response [24]. The results revealed SOS induction in the *optA1 gpt* strain starting at 4 hours after purine removal (Fig. 5A). This time coincides with the culture reaching its maximum value of nucleoid complexity (see Fig. 3C) and the start of the decline in culture viability (Fig. 3A).

We also studied the effect of *recA* and *sulA* deficiencies on the survival of the *optA1 gpt* strain. These mutations produced opposite effects (Fig. 5B): the *recA* defect dramatically sensitized the cells, leading to an immediate viability loss after Hx removal, very similar to the rapid early death of *recA* strains during TLD [5], [8]. In contrast, the *sulA* defect alleviated lethality (Fig. 5B), indicating that SOS-mediated lethal filamentation is a contributing factor to cell death [5].

### dGTP starvation on solid media

**Figure 6:**
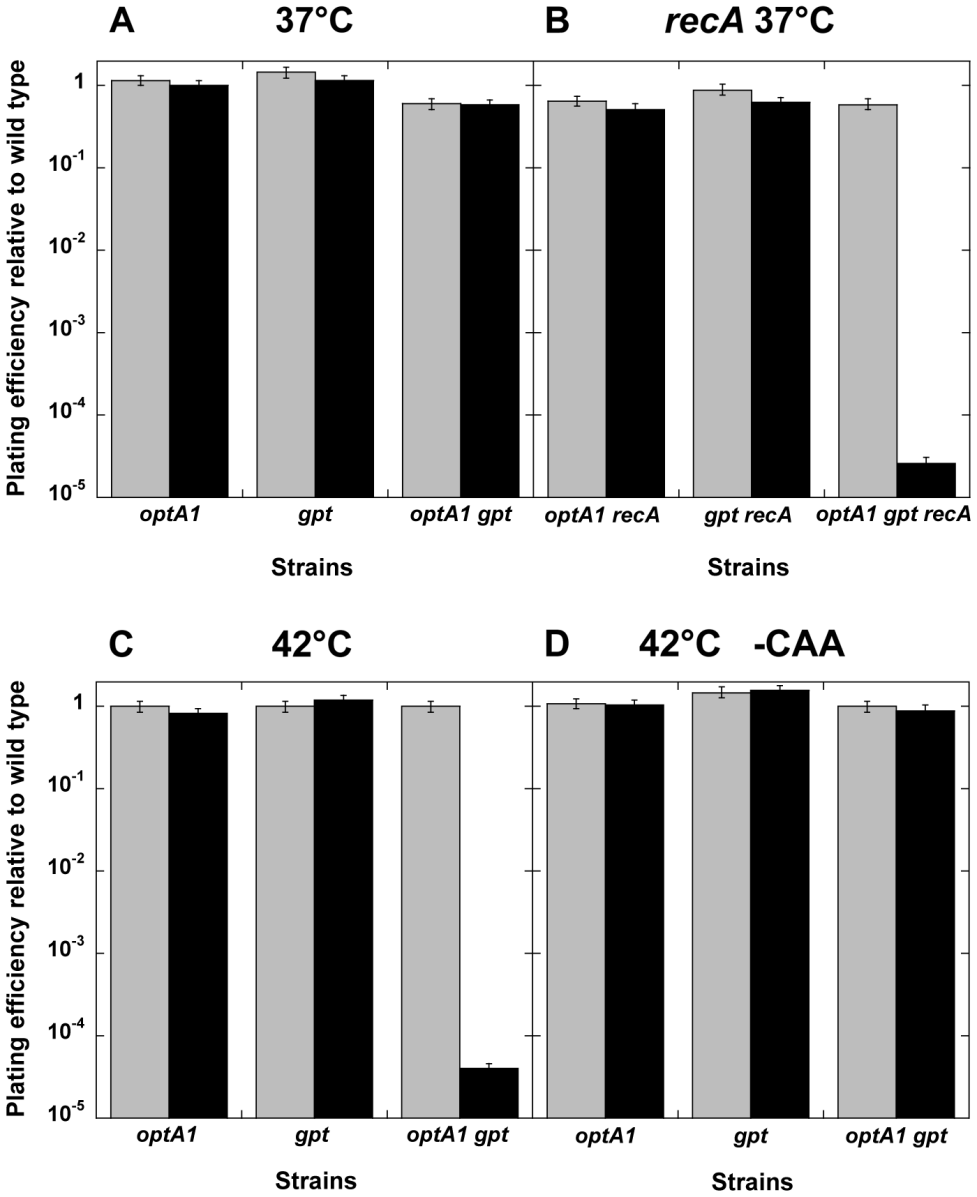
Plating efficiency of *optA1 gpt* strains on solid media with or without hypoxanthine. The indicated strains were grown overnight in minimal medium with CAA in the presence of hypoxanthine (Hx), and a series of 10-fold sequential dilutions were plated on minimal media plates supplemented with CAA, with (grey) or without (black) Hx, to determine the plating efficiency. Plates were incubated at the indicated temperature on glucose medium supplemented with CAA (**A, B, C**) or without CAA (**D**). Standard deviations (error bars) were calculated from three experiments.

Another informative aspect of dGTP starvation was revealed from plating efficiency determinations on solid media. In these experiments, the *optA1 gpt* strain was first grown to saturation in medium containing hypoxanthine, followed by plating on medium lacking hypoxanthine. It was observed that the *optA1 gpt* strain was able to form colonies on these plates with normal efficiency, at least when the plates were placed at 37° (Fig. 6A). In contrast, a strongly reduced plating efficiency (10^−4^ or less) was observed when the plates were placed at 42° (Fig. 6C). This loss of plating efficiency required the presence of casamino acids (CAA) (Fig. 6D). The more sensitive *optA1 gpt recA* triple mutant strain was not able to produce colonies, even at 37° (Fig. 6B). Overall, these results are consistent with the requirement for maintenance of an active growth status for the *optA1 gpt* strains. Presumably, in developing colonies on the plate the growth rate can be sufficiently slowed down, perhaps due to nutrient limitation in the colony environment, to permit survival. Such escape from death is apparently not possible at 42°C, probably due to increased origin firings at this higher temperature [25]. No escape is possible at any temperature for the Δ*recA* strain. The surviving fraction of cells (10^−5^ to 10^−4^) observed on these plates also reflects the appearance of suppressor mutants, as described below.

### Suppressor mutants of dGTP starvation

The starvation experiments in the liquid media show an apparent recovery of the *optA1 gpt* and *optA1 gpt recA* cultures after some 6–8 hrs (Figs. 3A and 5B). In fact, fully-grown cultures can be obtained in many cases after overnight growth. When these fully-grown cultures were diluted and subjected to a repeat starvation procedure, the cells proved resistant. We concluded that they had incurred suppressor mutations rendering the cells resistant. We also investigated the colonies that appeared on the 42°C glucose+CAA solid media plates of Fig. 6. When testing several of these colonies, it was found that they, too, were resistant to subsequent starvation, indicating that they had acquired mutations that allowed them to escape death.

The plating experiment of Fig. 6C proved a convenient avenue for obtaining a large number of resistant clones for further analysis. A total 108 colonies (from 10 independently grown cultures) were picked from the restrictive plates, purified and subjected to a repeat growth and plating cycle. Out of the 108 clones, 106 proved fully resistant. One obvious way by which resistance could be acquired is loss of the OptA1 phenotype through inactivation of the *dgt* gene. We tested for the possible loss of the OptA1 phenotype using the bacteriophage T4 assay (see Materials and Methods). This revealed that 20 out of the 106 clones (∼20%) had lost the OptA1 phenotype, likely due to loss of *dgt* function. The remaining 80% presumably represent mutants that lost some other functions involved in nucleotide metabolism (for example *purR*, see Fig. 1A) or in aspects of DNA replication and/or recombination. Further study of these suppressors may prove informative regarding the underlying mechanisms.

**Figure 7:**
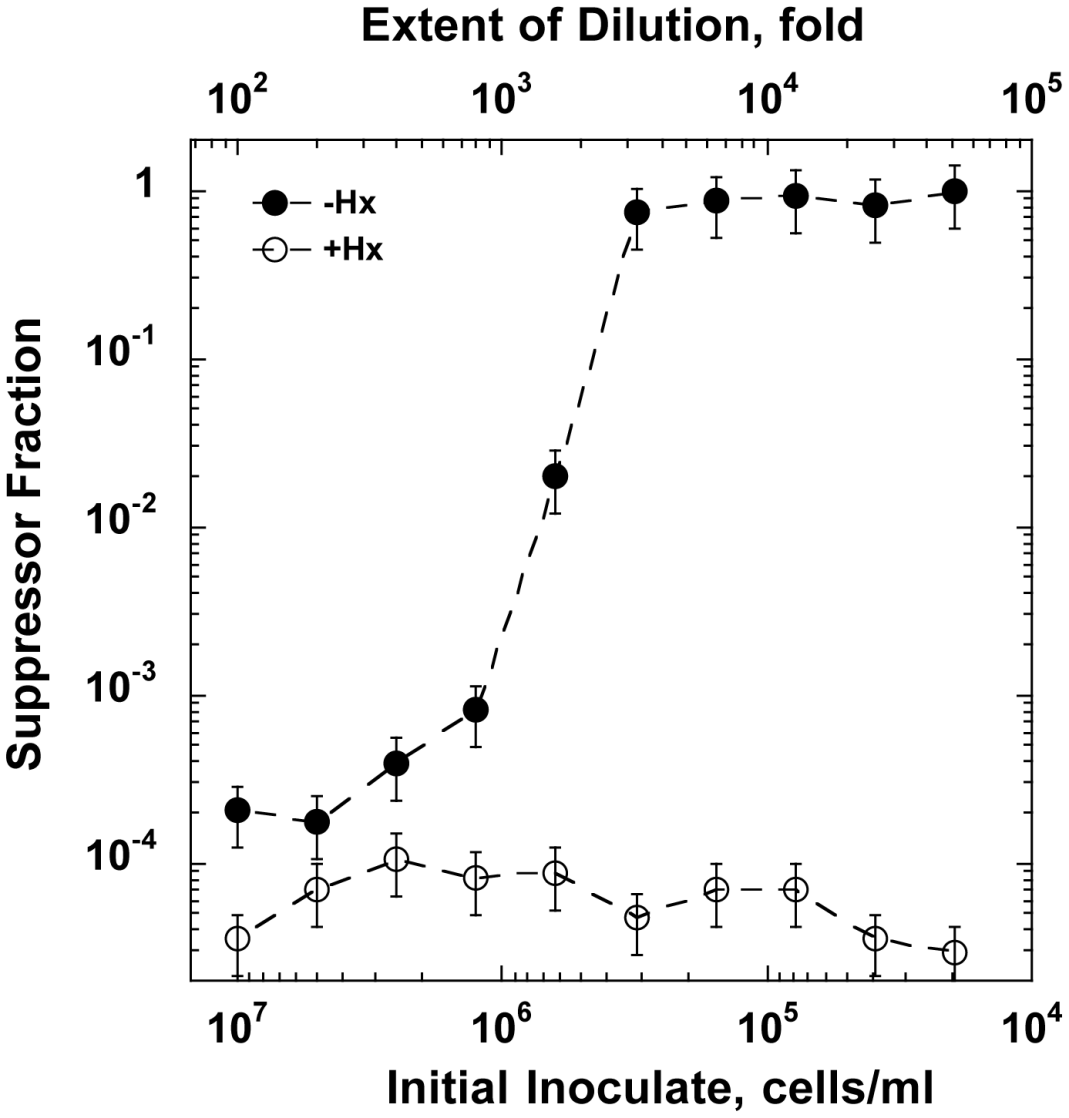
The outcome of dGTP starvation experiments for cultures initiated at decreasing cell densities. A stationary phase *optA1 gpt* culture was diluted to the indicated extents into minimal medium with casamino acids with hypoxanthine (Hx) (open symbols) or without Hx (closed symbols). The resulting cultures were subjected to overnight growth at 37°C, and samples of the resulting cultures were analyzed for the presence of suppressors resistant to subsequent repeat starvation using the tests described in Fig. 6C. The results are presented as the % suppressors observed. Assuming, based on the data of Figs. 1 and 3, that an about 100-fold increase in OD is needed from the beginning of starvation to reach lethal status, the critical biomass density (midpoint value) for cellular adaptation can be roughly estimated. At an inoculate value of 5×10^5^ cells for the midpoint and an experimentally determined correlation between OD_630 nm_ and cell titer (3.3×10^8^ cells/ml per OD_630 nm_), a 100-fold OD_630 nm_ increase would correspond to 100×(5×10^5^)/(3.3×10^8^) = 0.15.

One additional experiment (Fig. 7) provided useful insight into the emergence of suppressors along with confirming the critical importance of cell or biomass density (biomass density is a more accurate term here because cells progressively filament during starvation). In this experiment, a non-starved stationary culture was diluted to different extents - over a range of three orders of magnitude - and inoculated into media with or without hypoxanthine for an overnight growth attempt. The results showed that all cultures were able to reach full-grown density after overnight growth. However, when analyzed for the presence of suppressors, the cultures differed dramatically depending on their starting dilution. For cultures started at low to modest dilutions (100- to 800-fold) the majority of cells in the final culture had remained sensitive to dGTP starvation. On the other hand, dilutions of 3,000-fold or larger yielded cultures composed entirely of resistant clones (Fig. 7). Thus, relatively high densities permit survival without mutation, likely by reduction in growth rate, whereas low densities produce apparent survival only by mutation.

## Discussion

In this work, we have characterized a novel type of replication stress caused by specific starvation for the DNA precursor dGTP, which leads to growth impairment and cell death. This phenomenon, which we have called dGTP starvation, shares features with the previously described phenomenon of thymine-less death (TLD), which has been investigated for many decades. In TLD, specific starvation is for the DNA precursor dTTP, and current models of TLD have focused on the impairment of DNA synthesis by this lack of dTTP. If correct, starvation for any single dNTP is predicted to have the same or similar consequences. Our present study addresses this critical point, which could not be addressed previously due to the lack of means to specifically starve cells for any dNTP other than dTTP. In addition, our present results can be used to take issue with certain other models for TLD focused on the uniqueness of thymine nucleotides, perhaps related to the synthesis of cell wall components or other essential cell compounds [26]. Overall, we note similarities and dissimilarities with TLD, which we will summarize below.

### The properties of dGTP-starved strains

Our investigation of the hypoxanthine-starved *optA1 gpt* strains can be summarized by the following findings:

- Rapidly growing *optA1 gpt* cells dramatically slow down their division rate when deprived of hypoxanthine and manage to make only about two additional doublings over the next several hours (Fig. 3A).
- Subsequently, they show loss of viability, which is extensive and only appears to be stopped by the appearance of resistant variants (suppressor mutants).
- A recombination-deficient Δ*recA* strain is not able to show any additional growth upon hypoxanthine deprivation and loses viability immediately (Fig. 5B).
- The starved cells suffer from filamentation, accompanied by the appearance of cellular bulges containing expanded nucleoids (Fig. 2), likely reflecting high complexity genomes that cannot be untangled.
- The starved cells suffer from a rapid, specific dGTP depletion (Fig. 4D), which results in an immediate cessation of DNA synthesis (Fig. 3E).
- At later times, a modest recovery of the dGTP concentration occurs, accompanied by a corresponding recovery of the DNA synthesis capacity. However, neither the dGTP level nor the DNA synthesis capacity are able to reach the pre-starvation levels.
- An increased ori/ter ratio is observed, which we interpret to indicate continued creation of new replication forks at OriC along with stalled or slowed down forks moving towards the replication terminus (Fig. 3C).
- Induction of the SOS response, reflecting a significant level of DNA damage (Fig. 5A).
- The appearance of suppressors that are able to escape the starvation protocol and represent starvation-resistant mutants (Figs. 6 and 7).

Each of the findings (a-h) above can find their counterpoint in the phenomenology of TLD, although quantitative differences are certainly noted. This similarity of observations supports our contention that TLD and dGTP starvation are, in fact, two manifestations of the same underlying phenomenon, in which cells deprived of a single DNA precursor suffer from chromosomal distress that ultimately kills them.

We conclude that in dGTP-starved cells, like in TLD, ongoing replication forks are slowed down and even stall (Fig. 3E) due to the deprivation of one of the DNA precursors. The deprivation of dGTP is seen in dramatic fashion immediately upon hypoxanthine withdrawal, where both dGTP level and DNA synthesis rate are reduced to near zero (Figs. 3E, 4D), but also later when, despite a modest recovery, dGTP levels remain reduced and continue to decline. At the same time, the nutritional status of the cells remains high, leading to continued high levels of RNA and protein synthesis, as is also clear from the about 100-fold increase in biomass during the first few hours of starvation (Fig. 3A). Continued biomass growth permits continued initiations of new forks at the chromosomal origin (oriC). The slowdown of DNA synthesis along with continued new initiations is supported by the observed increase in the ori/ter ratio (Fig. 3C). The resulting build-up of chromosomal complexity (Fig. 3C) then leads to a series of secondary consequences, such as replication forks collisions, DNA bulges reflecting unresolved complex chromosomes (Fig. 2D and E), double-stranded breaks, SOS induction (Fig. 5A), and lethal filamentation (Fig. 2), as also occurring in TLD. The occurrence of double-strand breaks and the need for their repair is clearly suggested by the exquisite sensitivity of the *recA*-deficient *optA1 gpt* strain to dGTP starvation (Figs. 5B, 6B). Survival of the cells during the early cessation of DNA synthesis (Figs. 3E, 4D) appears to be critically dependent on recombinational repair, indicating that double-strand breaks are occurring at this stage [27]. This fully resembles the sensitization of *recA* mutants to the early stages of the TLD process [8], [9]. While in *rec* ^+^ cells the damaged forks can be repaired, their repair does not solve the stalling of the forks as long as the dGTP concentration stays limiting. It is likely that this early stalling is an important contributor to the build-up of chromosome complexity (ori/ter ratio) that takes place over the next several hours, culminating in SOS induction, filamentation, and cell death at the later time (4–6 h).

### Differences between dGTP starvation and TLD

At the same time, differences between dGTP starvation and TLD can be noted. Quantitatively, TLD appears to be a more destructive phenomenon, causing a more immediate loss of colony-forming ability (three orders of magnitude during three hours [2], [5], [8], [9]. During dGTP starvation, we observed only an ∼1.5 order of magnitude decline after the initial phase of continued cell divisions (Fig. 3A). Thus the effect of dGTP starvation is milder than that of TLD. The important distinction is likely that in TLD the necessary precursor thymine is experimentally totally absent [26], [28], while the cells have no avenue to synthesize dTTP by alternative means. In contrast in dGTP starvation, dGTP can still be produced, albeit at limited levels.

It might be suggested that dGTP starvation is more comparable to the phenomenon of thymine limitation, where *thyA* cells are grown at low, rate-limiting thymine concentrations while displaying an increased ori/ter ratio [29]. However, dGTP starvation differs from this type of limitation in one critical aspect: thymine-limited strains grow indefinitely in a steady state [30], i.e., the optical density, DNA concentration, and colony forming units increase exponentially at the same rate provided that the external thymine concentration is above the minimal required according to strain's background. In the case of dGTP starvation, cells are definitely not in steady state. For example, the increase in biomass until the arrest (about 100-fold) is not matched by that of the viable cell count (about 4-fold) (Fig. 3A). This discrepancy between biomass and cell count is consistent with the observed filamentation of the dGTP-starved cells (Fig. 2). Also, during thymine limitation no cell death is observed. Kinetically, the initial rapid disappearance of dGTP (Fig. 4D) along with the associated cessation of replication fork movement (Fig. 3E) places dGTP starvation phenomenologically closer to dTTP starvation than to thymine limitation.

dGTP starvation differs from TLD in that our experiments show a recovery of dGTP concentration at later time points. This recovery is likely a transcriptional response to the hypoxanthine withdrawal, which has no equivalent for dTTP production in TLD. Nevertheless, the recovery of dGTP is insufficient and dGTP levels continue to decline from that point on. Also note that the reduced DNA synthesis rate measured at later times is to be distributed over an increased number of forks. Thus, the rate of progression for each individual fork will be reduced accordingly. A rough estimate suggests that the 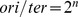 rate per fork may be down at least one order of magnitude. At such reduced rates, lethal chromosomal complexity may not be avoidable.

A third difference between dGTP starvation and TLD is that we did not find evidence for extensive origin destruction. Origin destruction has been discovered as one of the later aspects of TLD, purportedly by RecA-mediated ‘repair’ of multi-forked oriC [6], [9]. Presumably, in the presence of low dGTP concentrations, enough DNA synthesis can take place to avoid this step of origin destruction, although it does not prevent cell death. Interestingly, during dGTP starvation, Δ*recA* strains die at an accelerated rate that is very similar to the death rate of Δ*recA* strain during TLD (∼5% survival after 2 h of starvation) (Fig. 5B and [8], [9]). In both examples, no origin destruction occurs [9]. In this case, efficient killing occurs by events away from the origin, likely by lack of repair of stalled and broken replication forks.

### Nucleoid complexity during dGTP starvation

Nucleoids with increased complexity occur normally in bacteria under conditions of fast growth. Under these conditions, the generation time (τ) is shorter (faster) than the C-time (time needed to complete a round of chromosomal synthesis), which is achieved by the firing of newly replicated origins prior to completion of the previous rounds, resulting in more complex chromosomes [31]. On the other hand, it appears that in wild-type cells the *C*-time never is never greater than twice the lowest achievable doubling time (*C*≤2*τ*), so that the number of ongoing replication rounds (or ‘fork positions’, *n* = *C*/*τ* [32]), a quantitative measure of nucleoid complexity, rarely exceeds 2. Conditions of *n*>2 have been obtained experimentally upon severe thymine limitation of *thyA* strains at very low extracellular thymine concentrations [29], [33]. Under such conditions, cells continue to increase their size, culminating in distorted, monstrous shapes [34], resembling the ones observed in our study (Fig. 2). Due to the deviation from steady-state growth, a physiological characterization of such distorted cells is difficult, and the same reason prevents a precise calculation of *C* and *τ* during dGTP starvation. However, based on our measured ori/ter ratios it is possible to estimate *n* using the equation 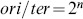 [22]. During dGTP starvation, the ori/ter ratio reaches a value of near 8, indicating a value of *n* near 3.0 (Fig. 3C). This would indicate the presence of a total of 14 (2+4+8) active forks per chromosome (see Fig. 3C). Extreme chromosome complexity due to overinitiation in a *dnaA* overexpressor strain has been shown to lead to collapse of replication forks, collisions between adjacent forks, and lethal chromosomal damage [35]. The existence of a limit to the extent of nucleoid complexity was proposed by Zaritsky [36] in his *Eclipse* model (this term was adopted from the Nordström studies on plasmid R1 replication [37]), which states that, due to structural constraints, scheduled initiations normally fire only if the previous fork has moved away some minimal distance from the origin. If this critical condition is not met, the scheduled initiation is postponed to avoid collisions of replication forks that may endanger the integrity of DNA [36]. One might speculate that under the starvation conditions discussed here this minimal distance limit is breached.

### Genetic change versus adaptation during dGTP starvation

Another, informative distinction between dGTP starvation and TLD is the recovery of the dGTP-starved liquid cultures after about 7 h (Fig. 3A). As described in the Results, recovery in this experiment is due to growth of mutants that have become resistant to the starvation condition. No production of resistant mutants occurs during TLD. This distinction between the two starvation procedures undoubtedly results from the fact that in TLD there is a zero provided supply of dTTP precursors, combined with the fact that the *thyA* cells do not have access to any alternative pathway by which dTTP might be synthesized, while during dGTP starvation there is a restricted, but not necessarily zero dGTP supply. Investigation of the suppressors of dGTP starvation may provide new insights into the various metabolic processes that can affect the ability of the cells to survive the dGTP-restricted conditions. We already noted that one category of suppressors is deduced to map in the *dgt* gene (∼20%), which is expected to restore dGTP levels. The majority of suppressors however reside at other loci, and their nature remains to be determined. It is an interesting question whether the suppressors represent preexisting mutants present in the population prior to initiation of the starvation conditions, or whether they are generated during the limited growth permitted during the starvation conditions. In view of the dNTP pool imbalances generated due to the dGTP drop, DNA replication during these conditions is likely to have reduced fidelity [38], [39]. Possibly such reduced fidelity may account for the relatively high frequency (10^−4^ to 10^−5^) of suppressors found in the plating experiments of Fig. 6.

As an alternative to genetic mutation, cells can survive dGTP starvation by entering a slower-growth phase in which origin firing is reduced to be compatible with the newly established slow DNA synthesis rate (Figs. 6, S1). This is clearly evidenced by the dilution experiments (Fig. S1), where increased cell densities slow down growth and permit survival. A further example of this type of survival is provided by the plating-efficiency of the *optA1 gpt* strain on solid media lacking hypoxanthine (Fig. 6). When plated at 37°C, the cells are able to develop into colonies, but at 42°C this is not the case; instead, suppressor colonies appear, at a frequency of 10^−5^ to 10^−4^. Inside developing colonies, growth may be slowed down sufficiently to allow cells to reach an adaptive phase, and this may be the case at 37°C, but not at 42°C, where increased metabolic activity, such as increased origin firings [25] may push the cells over the edge of sustainability. Conversely, reduction in origin firings by omitting the casamino acid supplement (CAA) from the plates allows cells to survive even at 42°C (Fig. 6D).

These growth-dependent effects described above are not unique to dGTP starvation. For example, even in TLD, growth-dependent effects have been described. While in TLD no growth on plates lacking thymine occurs (as thymine is an essential compound and *thyA* strains do not posses any alternative ways for dTTP synthesis), the extent of TLD can, nevertheless, be moderated by changes in growth conditions. For example, immunity to TLD can be provided by inhibition of protein or RNA synthesis [40], [41] or by the silencing of new initiations in *dnaA*(Ts) strains [42]. In fact, nutritional shift-up of certain *thyA* strains can promote death even in the presence of thymine, presumably due to a newly created imbalance between the rates of origin firing and DNA synthesis [43].

### Some perspectives

The discovery and analysis of the phenomenon of dGTP starvation solves one of the outstanding questions regarding TLD since it was discovered some six decades ago: whether the phenomenon is thymine-specific or whether it can be provoked by starvation for other DNA building blocks. Indeed, even though the proposed mechanisms for the inactivation of *thyA* strains during TLD have become increasingly substantiated, it is still important to clearly separate the killing process from the thymine specificity. The phenomenon of dGTP starvation solves this basic issue: starvation for other DNA precursors (like dGTP) should be equally lethal.

The finding that a critical starvation for the DNA precursor dGTP can cause cell death may lead to additional avenues for therapeutic applications. The dGTP model as described here may present a particularly realistic model system for cell death in such applications. In such cases the affected nucleotide may become critically restricted as presented here but not completely absent as in the TLD model system. dGTP may also be an attractive target as it has typically the lowest concentration among the four DNA precursors [44]. Interestingly, human cells have been found to contain a novel dGTPase, termed SAMHD1, that has properties similar to the bacterial Dgt enzyme: it likewise hydrolyzes dGTP to yield deoxyguanosine and triphosphate [45], [46]. SAMHD1 activity has been shown to act like a viral restriction factor in cells where it is expressed at elevated levels by lowering the dNTP concentrations sufficiently so that viral entities like HIV-1 cannot replicate [45], [46]. SAMHD1 also protects the cells against autoimmune responses, such as the Aicardi-Goutieres syndrome [45]. While in those cases SAMHD1 acts like a restriction factor by inhibiting viral replication and creating conditions that lead to the breakdown of a variety of RNA and DNA substrates, it is imaginable that this activity under certain physiological conditions in actively growing cells could also be directed towards the cellular DNA.

## Materials and Methods

### Bacterial strain constructions

All shown experiments used *E. coli* strain MG1655 and its derivatives. Genetic deficiencies were introduced into MG1655 by P1 transduction using P1*virA*. The *optA1* allele of *dgt* was introduced linked to transposon *zad-220*::Tn*10* as described [43]. The *gpt*::*kan* allele was obtained from the National BioResource Project (NIG) of Japan (http://www.shigen.nig.ac.jp/ecoli/strain/top/top.jsp). The p*urR*::*cat*, *recA*::*cat* and *sulA*::*cat* mutants were generated by the method of Datsenko and Wanner [47] using primers described in Table 1. For testing SOS induction, the relevant strains were transformed with plasmid pSK1002, which contains the *lacZ* reporter gene fused to the *umuDC* promoter [24].

**Table 1:** Primers used for construction of chromosomal gene deletions.

Target gene	Pairs of primers (5′-3′)	Template
*purR*	ATGGCAACAATAAAAGATGTAGCGAAACGAGCAAACGTTTCCACTACAACGTGTAGGCTGGAGCTGCTTC TTAACGACGATAGTCGCGGAACGGGCCGTCAGCCACGGAGCGGCGTTCAACATATGAATATCCTCCTTAG	pKD3
*recA*	ATGGCTATCGACGAAAACAAACAGAAAGCGTTGGCGGCAGCACTGGGCCAGTGTAGGCTGGAGCTGCTTC TTAAAAATCTTCGTTAGTTTCTGCTACGCCTTCGCTATCATCTACAGAGACATATGAATATCCTCCTTAG	pKD3
*sulA*	ATGTACACTTCAGGCTATGCACATCGTTCTTCGTCGTTCTCATCCGCAGCGTGTAGGCTGGAGCTGCTTC TTAATGATACAAATTAGAGTGAATTTTTAGCCCGGAAAGTTGTCTCGTGGCATATGAATATCCTCCTTAG	pKD3

### Media and growth

For strain construction, maintenance, and determination of viable counts, LB medium was used with supplementation of the following antibiotics, where appropriate: tetracycline (15 µg/ml) for *optA1* linked with *zad-22*::Tn*10*, kanamycin (50 µg/ml) for *gpt*::*kan*, chloramphenicol (25 µg/ml) for the *purR*::*cat*, *recA*::*cat* and *sulA*::*cat* alleles, and ampicilin (100 µg/ml) for pSK1002 transformants. For experiments relating to starvation, cells were grown at 37°C in minimal medium containing Vogel-Bonner salts [48] containing glucose (0.4%), casamino acids (1%) (Becton-Dickinson), D-pantothenic acid (5 µM), and hypoxanthine (50 µg/ml). To assay the differential responses in media with or without purine source, two aliquots were filtered through a 25, 47 or 90-mm diameter polycarbonate membrane filter (0.4 µm pore size; Millipore) and diluted up to 10-fold in the identical medium with or without hypoxanthine (50 µg/ml). Aliquots for different assays were withdrawn at densities not exceeding 0.2 OD_630 nm_.

### Nucleotide extraction and HPLC analysis

Culture aliquots (300–350 ml) harvested at OD_630 nm_ = 0.2 were filtered through a 90-mm diameter polycarbonate membrane filter (0.4 µm pore size; Millipore). The filter was transferred to a Petri dish lid containing 10 ml of 60% aqueous methanol at **−**20**°**C. After 2 h at **−**20**°**C the filter was removed and the liquid suspension boiled for 5 min, followed by centrifugation for 15 min at 17,000× g and lyophilization of the supernatant. The residue was dissolved in 1 ml of sterile water, filtered through syringe filter (Millipore, 0.22 µm pore size) and lyophilized again. The final residue was dissolved 50 µl sterile water. HPLC analysis of the extracted dNTPs was performed by reversed-phase chromatography on an Agilent 1100 high-pressure liquid chromatography instrument with UV detection at 254 nm. Nucleotides were separated on a Zorbax Eclipse XDBC18 3.5 µM (150 by 4.6 mm) column equipped with a Zorbax Eclipse XDBC18 guard column, adapting a prior method used for the separation of nucleotides [38]. At a flow rate of 0.8 ml/min, a linear gradient of 70∶30 buffer A to buffer B was run to 40∶60 over 30 min. The gradient was then changed over 60 min from 40∶60∶0 to 0∶87.5∶12.5 for buffer A - buffer B - buffer C. To wash the column between samples the gradient was first changed from 0∶87.5∶12.5 to 0∶70∶30 over 10 minutes with a final stepwise change to 70∶30∶0 for an additional 20 min.

In a later set of experiments aimed at quantifying specifically dGTP during an extended starvation time course (Fig. 4D), a modified protocol was used, as follows. At a flow rate of 1 ml/min, a linear gradient of 75∶25 buffer A to buffer B was changed to 52∶48 over 23 min. The gradient was then changed over 12 min from 52∶48 to 49∶51 and for an additional 10 min from 49∶51 to 40∶60. To wash the column between samples the gradient was first changed from 40∶60∶0 to 0∶77.5∶22.5 for buffer A - buffer B - buffer C over 15 minutes and for an additional 10 min from 0∶77.5∶22.5 to 0∶70∶30 with a final stepwise change to 70∶30∶0 for an additional 10 min.

Buffer A consisted of 5 mM tetrabutyl ammonium phosphate (PicA Reagent; Waters), 10 mM KH_2_PO_4_, and 0.25% methanol adjusted to pH 6.9. Buffer B consisted of 5 mM tetrabutyl ammonium phosphate, 50 mM KH_2_PO_4_, and 30% methanol (pH 7.0). Buffer C was acetonitrile. Nucleotide standards were obtained from Sigma.

### DNA analysis

For chromosomal DNA extraction, 7- or 13-ml culture aliquots were harvested at various time points into the same volume of ice-cold PBS solution containing 20 mM NaN_3_. DNA extraction was performed with the Easy-DNA kit (Invitrogen) and quantitated by staining with Picogreen (Invitrogen). For determination of run-out DNA synthesis (Fig. 3D), rifampicin was added at time zero at a concentration 300 µg/ml. For ori/ter determination, the DNA was digested with *EcoR*I and subjected to Quantitative PCR (Stratagene Mx 3000) with SIBR Green detection using primers (Table 2) specific to the origin and the terminus regions of the *E. coli* chromosome [49].

**Table 2:** Primers specific to the origin (ori) and the terminus (ter) regions of the *E. coli* chromosome [49] used for ori/ter ratio determination using Quantitative PCR.

Region	Pairs of primers (5′-3′)
ori	GAGAATATGGCGTACCAGCA AAGACGCAGGTATTTCGCTT
ter	TCCTCGCTGTTTGTCATCTT GGTCTTGCTCGAATCCCTT

For determination of the DNA synthesis rate by pulse labeling, *optA1 gpt* cultures were grown with hypoxanthine to OD_630 nm_ 0.1, filtered, resuspended in the identical media with or without hypoxanthine, and brought to the same turbidity. Every 10–15 minutes 0.5 ml samples were withdrawn and pulse-labeled with 1 µCi of [methyl-^3^H]-thymidine at specific activity 0.5 µCi/nmole for 3 minutes. Samples were quenched with 0.5 ml of cold trichloroacetic acid (TCA 10%) containing 500 µg/ml of unlabeled thymidine to a final concentration 5% TCA and 250 µg/ml of unlabeled thymidine and kept on ice bath at least for 30 minutes. The entire samples were then collected on pre-wet 25-mm glass microfibre filters (Whatman), and washed with cold TCA (5% with 250 µg/ml of unlabeled thymidine) and 100% ethanol. The radioactivity on the filters was determined in a LS6500 liquid scintillation counter (Beckman) with Ecolume liquid scintillation cocktail (MP Biomedicals).

### Microscopy

Aliquots of growing cultures were fixed with 0.25% formaldehyde and stained with DAPI. The cells were visualized by Nomarsky and DAPI fluorescence microscopy (NIKON eclipse E600) and photographed using a Micropublisher CCD color Camera (QImaging). Live/Dead stain (Invitrogen) was used to test viability of the cells.

### Liquid β-galactosidase assay

5.0-ml samples were removed, and the cells were pelleted in a microcentrifuge. β-Galactosidase assays were performed essentially as described by Miller [18]. Cell pellets were resuspended in 1 ml of Z-buffer (60 mM Na_2_HPO_4_, 40 mM NaH_2_PO_4_, 10 mM KCl, 1 mM MgSO_4_, 10 mM dithiothreitol). 80 µl of chloroform and 40 µl of 0.1% sodium dodecyl sulfate (SDS) were added to the cell suspension, which was then vortexed vigorously for 10 s. To start the reactions, 200 µl of ONPG (4 mM) was added, and the reaction mixtures were incubated at 30°C for 4.5 min. The reactions were stopped with 0.5 ml of 1 M sodium bicarbonate, and the cellular debris was pelleted. The optical density was recorded with a BECKMAN DU 640 spectrophotometer with a 405-nm filter. Miller units were calculated as follows: units = 1,000[(OD_405_/(t×v×OD_630 nm_)], where OD_405 nm_ denotes the optical density at 405 nm. OD_630 nm_ reflects the cell density at 630 nm, t is the reaction time in minutes, and v is the volume of culture used in the assay.

### Analysis of suppressor mutants

To test whether suppression of the sensitivity of the *optA1 gpt* strains to hypoxanthine deprivation resulted from loss of the *optA1* allele, a large number of clones that survived on the starvation plates at 42°C (see Fig. 6C) were tested for their ability to support growth of the bacteriophage T4 *tsL141* mutant [50], as described [43]. Clones that restrict growth of this phage at 30°C are *optA1* [51]. Wild-type phage T4D was used as a positive control. The T4 phages were obtained from Dr J.W. Drake, NIEHS.

## Supporting Information

Figure S1 Growth of an *optA1 gpt* strain in purine starvation medium when subjected to different dilutions. Cultures were grown exponentially in the presence of hypoxanthine (Hx) (50 µg/ml) as in the experiment of Fig. 3. At time 0 (OD_630 nm_ = 0.1), two aliquots were filtered and diluted 10-fold into identical fresh, prewarmed medium with (open circles) or without (black) Hx (first dilution). After two hours of growth, the cultures were diluted again 10-fold (circles), 25-fold (squares), or 50-fold (triangles) (second dilution). The y-axis values for turbidity (A) and viable count (B) are all normalized relative to the value at time zero, as explained in the Legend to Fig. 3A.
(PDF)

Table S1 (d)NTP pools in purine-starved strains. Nucleotide amounts were normalized relative to the ATP peak as in [38]. One unit corresponds to 8 nmole of nucleotide extracted from 50 ml of cell culture, harvested at 0.15 OD_630 nm_. ND, not detected (see main text and Fig. 4).
(DOCX)
