# Supplementary figures and images for: dGTP Starvation in Escherichia coli Provides New Insights into the Thymineless-Death Phenomenon
Source: PLoS Genet. 2014 May 8;10(5):e1004310. doi: 10.1371/journal.pgen.1004310 (PMC4014421; doi:10.1371/journal.pgen.1004310)

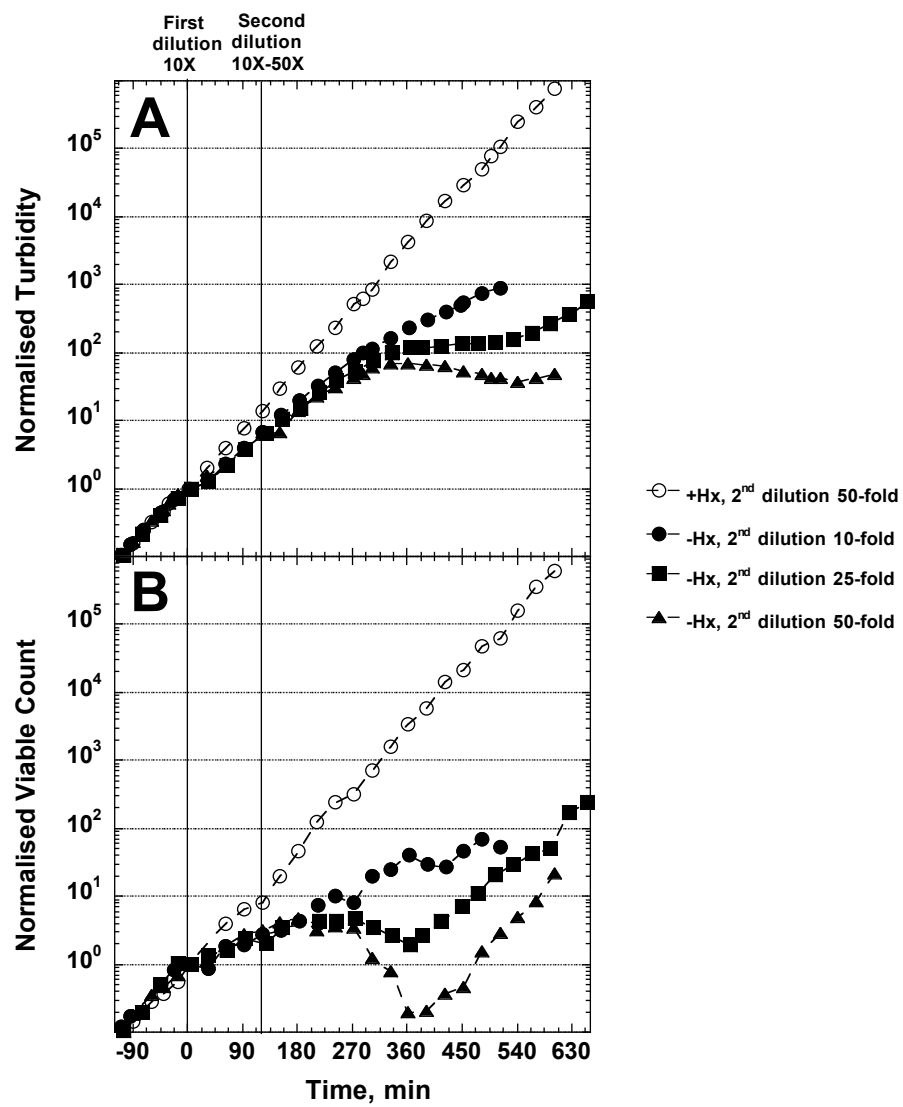

Supplement: Figure S1 — Growth of an optA1 gpt strain in purine starvation medium when subjected to different dilutions. Cultures were grown exponentially in the presence of hypoxanthine (Hx) (50 µg/ml) as in the experiment of Fig. 3. At time 0 (OD630 nm = 0.1), two aliquots were filtered and diluted 10-fold into identical fresh, prewarmed medium with (open circles) or without (black) Hx (first dilution). After two hours of growth, the cultures were diluted again 10-fold (circles), 25-fold (squares), or 50-fold (triangles) (second dilution). The y-axis values for turbidity (A) and viable count (B) are all normalized relative to the value at time zero, as explained in the Legend to Fig. 3A. (PDF) [file pgen.1004310.s001.pdf]
