# Supplementary material for: dGTP Starvation in Escherichia coli Provides New Insights into the Thymineless-Death Phenomenon
Source: PLoS Genet. 2014 May 8;10(5):e1004310. doi: 10.1371/journal.pgen.1004310 (PMC4014421; doi:10.1371/journal.pgen.1004310)
Supplement: Table S1 — (d)NTP pools in purine-starved strains. Nucleotide amounts were normalized relative to the ATP peak as in [38]. One unit corresponds to 8 nmole of nucleotide extracted from 50 ml of cell culture, harvested at 0.15 OD630 nm. ND, not detected (see main text and Fig. 4). (DOCX) [file pgen.1004310.s002.docx]

**Table S1** Nucleotide pool effects in purine-starved strains. Nucleotide amounts were normalized relative to the ATP peak as in [38].

|  | *optA1* | | | *gpt* | | | *optA1 gpt* | | |
| --- | --- | --- | --- | --- | --- | --- | --- | --- | --- |
|  | +Hx | -Hx(2h) | -Hx(4h) | +Hx | -Hx(2h) | -Hx(4h) | +Hx | -Hx(2h) | -Hx(4h) |
| dCTP | 0.053±0.003 | 0.056±0.002 | 0.053±0.003 | 0.046±0.002 | 0.052±0.004 | 0.048±0.004 | 0.059±0.003 | 0.094±0.008 | 0.078±0.009 |
| dATP | 0.051±0.003 | 0.050±0.002 | 0.050±0.002 | 0.061±0.003 | 0.051±0.004 | 0.056±0.002 | 0.050±0.003 | 0.059±0.01 | 0.059±0.007 |
| dTTP | 0.036±0.001 | 0.049±0.002 | 0.045±0.001 | 0.021±0.002 | 0.033±0.006 | 0.027±0.003 | 0.035±0.002 | 0.073±0.005 | 0.059±0.006 |
| dGTP | 0.016±0.003 | 0.020±0.005 | 0.018±0.006 | 0.027±0.002 | 0.038±0.019 | 0.032±0.004 | 0.018±0.002 | 0.003±0.001 | ND |
| ATP | 1.017±0.024 | 0.954±0.07 | 1.015±0.02 | 1.020±0.02 | 0.998±0.003 | 0.99±0.01 | 1.023±0.03 | 1.00±0.02 | 0.983±0.02 |
| UTP | 0.187±0.008 | 0.194±0.01 | 0.186±0.01 | 0.197±0.02 | 0.276±0.047 | 0.23±0.01 | 0.196±0.01 | 0.30±0.03 | 0.302±0.07 |
| GTP | 0.628±0.012 | 0.598±0.02 | 0.595±0.01 | 0.63±0.015 | 0.517±0.012 | 0.54±0.01 | 0.612±0.03 | 0.33±0.04 | 0.358±0.03 |

One Relative Unit corresponds to 8 nmole of the corresponding nucleotide extracted from 50 ml of the culture harvested at 0.15 OD_630nm_.

ND Not determined
